# Measuring Outcome in an Early Intervention Program for Toddlers with Autism Spectrum Disorder: Use of a Curriculum-Based Assessment

**DOI:** 10.1155/2014/964704

**Published:** 2014-03-10

**Authors:** Elizabeth C. Bacon, Sarah Dufek, Laura Schreibman, Aubyn C. Stahmer, Karen Pierce, Eric Courchesne

**Affiliations:** ^1^Department of Psychology, University of California, San Diego, 9500 Gilman Drive, La Jolla, CA 92093-0109, USA; ^2^Department of Psychiatry, Weill Cornell Medical College, 21 Bloomingdale Road, Rogers Building, White Plains, NY 10605, USA; ^3^Department of Psychiatry, University of California, San Diego, 9500 Gilman Drive, La Jolla, CA 92093, USA; ^4^Child and Adolescent Services Research Center and Autism Discovery Institute, Rady Children's Hospital San Diego, 3020 Children's Way, San Diego, CA 92123, USA; ^5^Department of Neurosciences, University of California, San Diego, 9500 Gilman Drive, La Jolla, CA 92093, USA

## Abstract

Measuring progress of children with autism spectrum disorder (ASD) during intervention programs is a challenge faced by researchers and clinicians. Typically, standardized assessments of child development are used within research settings to measure the effects of early intervention programs. However, the use of standardized assessments is not without limitations, including lack of sensitivity of some assessments to measure small or slow progress, testing constraints that may affect the child's performance, and the lack of information provided by the assessments that can be used to guide treatment planning. The utility of a curriculum-based assessment is discussed in comparison to the use of standardized assessments to measure child functioning and progress throughout an early intervention program for toddlers with risk for ASD. Scores derived from the curriculum-based assessment were positively correlated with standardized assessments, captured progress masked by standardized assessments, and early scores were predictive of later outcomes. These results support the use of a curriculum-based assessment as an additional and appropriate method for measuring child progress in an early intervention program. Further benefits of the use of curriculum-based measures for use within community settings are discussed.

## 1. Introduction

Advancements in the identification, diagnosis, and treatment of very young children with autism spectrum disorder (ASD) have challenged researchers and clinicians to examine alternative assessments of child progress and outcome in early intervention programs. The most common assessments evaluating change across developmental domains (i.e., cognition, communication, social skills, adaptive behavior, and behavior challenges) are standardized assessments [1–5]. However, several limitations to this type of measurement approach have been noted in the literature, including lack of sensitivity, testing constraints, and contextual issues [6, 7]. Furthermore, standardized assessments, in particular assessments of intelligence, are not as stable in toddlers and young children as they are at older ages [8]. Given the surge of early identification and early treatment programs, novel methods for tracking treatment progress are essential.

A recurrent theme throughout the intervention literature is the lack of sensitivity of standardized measures for children with ASD. For example, a child may show maintenance or decrease in standardized scores over time while simultaneously increasing in raw scores [9–13]. Discrepancy between standard scores and raw scores may occur because over time a child is expected to gain skills due to maturation. Therefore, when a child is not maintaining an expected rate of development, standardized scores can be affected negatively over time as the gap between chronological age and expected skills becomes larger as the child ages [14]. In the case of a developmentally delayed child (as is common in ASD), these children often are not progressing at the same pace as their typically developing peers. This may result in a decline in standardized scores, despite the absence of any actual regression and the presence of some progress. Thus, many children with ASD will still show measured impairment after, or experience slow progress during, intervention because standardized tests may not measure change in such small increments, masking progress made by the child [6]. While standardized assessments are useful for comparing groups of children receiving intervention and comparing progress of children with ASD in relation to typical development, they are not designed to measure the progress of an individual child in an intervention program. In addition, because standardized assessments measure broad ability level, they are not designed to be used for treatment planning and individualization. Often, more detailed information about progress toward specific goals is needed. As such, standardized assessments in isolation may not be the most informative tool to measure child progress during intervention.

Another concern related to use of standardized measures with children with ASD is the influence of constraints during test administration. Administration of standardized assessments is usually highly structured, with the intention of keeping the administration consistent across many participants and true to established norms. Due to these restrictions, it is likely that a child's behavior may be very different when assessed by an unfamiliar clinician in an unfamiliar clinic than during normal routines [9, 11]. Additionally, the lack of administration flexibility may be especially detrimental to the measurement of populations such as children with ASD, where lack of social motivation, attention deficits, and disruptive behaviors are frequently present. Koegel et al. [15] found improved scores on several assessments implemented with children with ASD when the clinician incorporated motivational and attentional strategies such as giving breaks to do preferred activities contingent upon compliance during testing. Koegel and colleagues [15] concluded that standardized assessments may be erroneously measuring test taking skills rather than actual abilities for the children in their study.

Ideally, child testing would provide valuable information that may be incorporated into an individual child's ongoing intervention program. However, standardized assessments were not designed to provide detailed information relating to treatment goal development. In fact, developing intervention goals based on a child's performance on standardized assessments is discouraged in order to avoid “teaching to the test.” Clinicians are advised to let at least six months pass before readministering the same standardized assessment to the same child as practice effects often are seen with multiple presentations of the same material within a recent time frame, making the results an invalid representation of the individual's abilities [16, 17]. The limitations of goal-specific information gathered from standardized testing combined with the restrictions regarding how often a test can be administered pose a problem for timely tracking of child progress and goal planning.

In order to address the issues discussed above, we propose that standardized testing be supplemented with the use of curriculum-based assessments to provide finer detail on child progress and to assist with treatment individualization and planning. This paper presents the results of an evaluation of the utility of the adapted student learning profile (aSLP) to measure progress of children in an early intervention program specific to the aSLP curriculum. The aSLP is a curriculum-based measure that assesses mastery of targeted skills to measure a child's progress during, and outcome after, an intervention program. The aSLP has the potential to measure child progress throughout ongoing intervention in a systematic way that better allows comparison of child progress and rate of learning in intervention within and across programs. We posit that using a combination of curriculum-based and standardized assessments to measure child functioning will provide a greater understanding of child progress and outcomes in early intervention.

## 2. Method

### 2.1. Participants

The toddlers and families in this investigation were participants in a larger multidisciplinary research project examining early neurobiological features and development of ASD at the University of California, San Diego. Toddlers at risk for an ASD were obtained from one of two sources: general community referral (e.g., website or outside agency) and a population-based screening method called the 1-Year Well-Baby Check-Up Approach [18]. Using this method, toddlers at risk for an ASD as young as 12 months were identified in pediatric offices with a broadband screening instrument, the Communication and Symbolic Behavior Scales-Developmental Profile Infant Toddler Checklist [19]. Toddlers were evaluated and tracked every six months until their third birthday when a final diagnosis was given. Experienced clinicians with expertise in the evaluation and diagnosis of ASD identified children at risk for an ASD by incorporating criteria for ASD on the Autism Diagnostic Observation Schedule [20] along with the clinician's clinical judgment. Child participants who were determined to be at risk for ASD were offered intervention at a university-affiliated clinical research program. Seventy-two families were referred for intervention, and 49 families chose to receive treatment at our program. Of the 49 children who received treatment from our program, 45 of the children (mean age = 22.67 months, *r* = 13–27 months) were included in the analyses. Inclusion criteria included completion of six months or more of early intervention treatment as well as completion of diagnostic and cognitive assessments prior to and at the end of the intervention program. Three children were excluded because they received less than six months of treatment; one child was excluded because of parental failure to complete assessments after intervention. Additional participant demographic information can be found in Table 1. Forty-two of the 45 children continued to meet criteria for an ASD upon exit of the intervention program while three children did not.

### 2.2. Early Intervention Program

#### 2.2.1. Curriculum

The Strategies for Teaching Based on Autism Research (STAR) curriculum [21] was used as the basis for the early intervention in-home programming. The STAR program is a comprehensive behavioral intervention program with a curriculum designed specifically for children with ASD and includes instructional strategies of Discrete Trial Training [22–24], Pivotal Response Training [25, 26], and teaching in Functional Routines [27, 28]. In an effort to improve the developmental appropriateness of the curriculum for these very young children, the STAR curriculum was augmented with developmental approaches applied through Teaching Social Communication (TSC) to Children with Autism. TSC is a manualized curriculum developed by Ingersoll and Dvortcsak [29] used to target social-communication goals in young children with ASD. TSC focuses on the relationship between adult responsivity and children's social-communicative development. In the TSC curriculum, an early childhood interventionist (ECI) combines naturalistic behavioral strategies and developmental strategies. For example, the interventionist would respond to all communicative attempts by the child as if they were purposeful and recast expanded communication to facilitate communicative growth.

#### 2.2.2. Treatment Delivery

Each child received approximately 6–12 hours per week of direct one-on-one intervention with a trained ECI at home until 36 months of age (*M* = 9.044; *r* = 4.5–12). ECIs were bachelor's degree or undergraduate-level research assistants with previous experience with young children with ASD. Each ECI received extensive didactic and hands-on training in behavioral principles and the STAR and TSC programs discussed above. Fidelity of implementation was reached for each intervention strategy as determined by using all components of the intervention correctly at least 80% of the time across two different children. Programs were developed and supervised by master's degree-level clinicians (i.e., in-home coordinator) experienced in ASD, with oversight from two doctorate-level clinical psychologists with extensive experience in early behavioral intervention for this population. In addition, parent training was provided throughout the course of participation. As the focus of this paper is on the evaluation of a curriculum-based measure to assess children during intervention, details regarding the assessments rather than the intervention are emphasized. However, additional details regarding the intervention provided can be obtained from the authors.

### 2.3. Measures

#### 2.3.1. The Adapted Student Learning Profile

The aSLP is a curriculum-based assessment for determining student learning goals and was adapted from the STAR curriculum to include additional goals from the TSC curriculum (see [21, 29]; e.g., Table 2). The aSLP provides an extensive list of skills targeted in the STAR and TSC curricula and allows for the assessor to indicate the child's performance level on each skill across six domains, receptive language, expressive language, spontaneous language, functional routines, preacademic concepts, and play and social interaction concepts. Data were analyzed using overall scores only for ease of analysis. The aSLP is administered by presenting each item up to five times to the child and observing the child's response. This is conducted in a structured format, and no teaching was done during the assessment. The assessor then rates the child's response, indicating if the child did not demonstrate the skill or showed partial demonstration of the skill or mastery of the skill (e.g., see Table 2). The entire aSLP takes about 30–45 minutes to complete. Each child's in-home coordinator completed an aSLP at intake and every three months thereafter to determine performance and progress. This method of measurement resulted in a variable number of aSLP assessments across children depending on how long they participated in the early intervention program (*M* = 4.53, *r* = 2–8 aSLPs). All available scores were used to examine learning trajectories. All toddlers had aSLP scores at intake into the program and at age three when they exited the program. Scores on the aSLP reflected child performance on items related to the six domains. The amount of progress made during intervention was quantified by taking the aSLP score at exit and subtracting the aSLP score at intake, yielding a change score for each child (aSLP exit score minus aSLP intake score).

#### 2.3.2. Mullen Scales of Early Learning (MSEL)

The MSEL assesses developmental functioning of children between birth and 68 months [30]. An examiner measures child functioning level through a series of play-like tasks over five domains, gross motor, fine motor, receptive language, expressive language, and visual reception skills. Children were assessed on the MSEL at intake and exit from the program. For each scale, the assessment derives a T-score with a mean of 50 and standard deviation of 10, a percentile score, and an age equivalent indicating at what developmental age the child is performing. An early learning composite (ELC) score is calculated from the total of scores on all scales (excepting the gross motor scale) with a mean of 100 and standard deviation of 15. Change on the MSEL while in intervention was calculated using changes on the ELC from intake to exit from the program (MSEL ELC Exit Score minus MSEL ELC Intake Score).

#### 2.3.3. Wechsler Preschool and Primary Scale of Intelligence, 3rd Edition (WPPSI)

Three children were administered the WPPSI rather than the MSEL at exit as these children were performing at ceiling levels on the MSEL at the close of their early intervention program. The WPPSI assesses cognitive development in children between two years, six months and seven years, three months [31]. It provides composite scores that represent intellectual functioning in verbal and performance domains as well as a full scale intelligence quotient (FSIQ) that represents a child's general intellectual ability. Each composite score has an average of 100 with a standard deviation of 15. For the three children who received the WPPSI, the FSIQ was used in lieu of the MSEL ELC score at exit.

#### 2.3.4. Vineland Adaptive Behavior Scales, 2nd Edition (VABS)

The VABS provides a measure of adaptive skills used to cope with challenges of daily living [32]. A caregiver completes a questionnaire regarding the individual's current level of functioning across five domains: communication, daily living skills, socialization, motor skills, and maladaptive behavior. The Vineland Scales are applicable to children with and without delays from birth to 18 years, 11 months. Standardization of the instrument included national samples of both “handicapped” and “nonhandicapped” children. All scales use standard scores with a mean of 100 and a standard deviation of 15, a percentile score, and an age equivalent indicating at what developmental age the individual is performing. Scores on all scales are combined to obtain an overall adaptive behavior composite (ABC) with a mean of 100 and a standard deviation of 15. Change on the VABS while in intervention program was quantified by calculating the changes on the ABC from intake to exit from the program (VABS ABC exit score minus VABS ABC intake score).

### 2.4. Statistical Analyses

Means with standard deviations were calculated for all assessments at intake into the program, exit, and change scores from intake to exit. In order to analyze whether the aSLP measured progress in intervention similarly to the MSEL and VABS, partial correlations were conducted controlling for the amount of time the child was in treatment. Lastly, a linear regression was conducted to analyze if aSLP scores at three months into intervention were predictive of aSLP scores at exit. This analysis was included to examine if early treatment progress on the aSLP is indicative of later outcomes, which would give practitioners information about the importance of early performance, and insight into the type of outcome that could be expected. Analyses were conducted using the IBM SPSS Statistics 22 statistical analysis package.

## 3. Results

**Figure 1:**
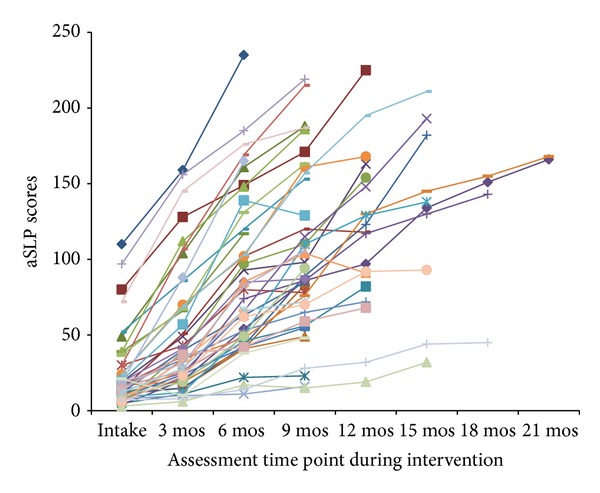
aSLP scores for each participant over time. Each participant is depicted by one line. The aSLP was administered to each child at intake into the early intervention program and every 3 months thereafter. The aSLP score depicts the number of skills mastered at each assessment. Significant changes were seen in aSLP scores from intake to exit from the early intervention program.

Results for all assessments are summarized in Table 3. Children showed variable rates and amount of progress as measured by the aSLP (see Figure 1). On average, participants entered the intervention program with 23.56 (*r* = 3–110) skills that were part of the curriculum and exited the program on average with a mastery of 120.58 skills (*r* = 16–235). A repeated measures *t*-test revealed significant differences in the number of skills measured on the aSLP between intake and exit from the program (*t*(44) = 12.778; *P* < 0.001). Children mastered an average of 97 (*r* = 8–187) skills while being in treatment. There was a very large range in scores with some children learning very few skills and others mastering many. Similar heterogeneity in scores was seen on the MSEL ELC (intake: *M* = 75.44, *r* = 44–115, exit: *M* = 83.51, *r* = 49–128; change scores: *M* = 8.07, *r* = −20–56) and VABS ABC (intake: *M* = 84.38, *r* = 65–130, exit: *M* = 83.13, *r* = 59–111; change scores: *M* = −1.24, *r* = −42–21). A repeated measures *t*-test revealed significant differences in MSEL scores at intake and exit (*t*(44) = 3.287, *P* = 0.002). There were no significant differences between intake and exit scores on the VABS.

### 3.1. Relationship between aSLP and Standardized Test Measures

**Figure 2:**
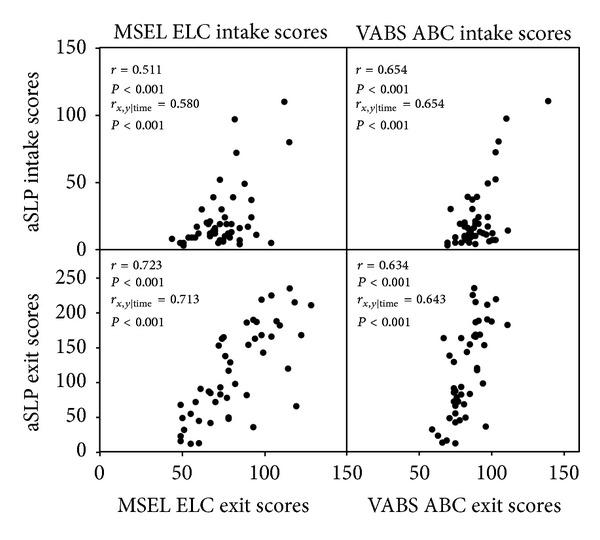
Correlations between the aSLP scores and MSEL ELC and VABS ABC at intake and exit of the early intervention program. Correlation coefficients are reported for Pearson correlations and partial correlations controlling for the amount of time in intervention. Significant positive correlations were found between all measures.

**Figure 3:**
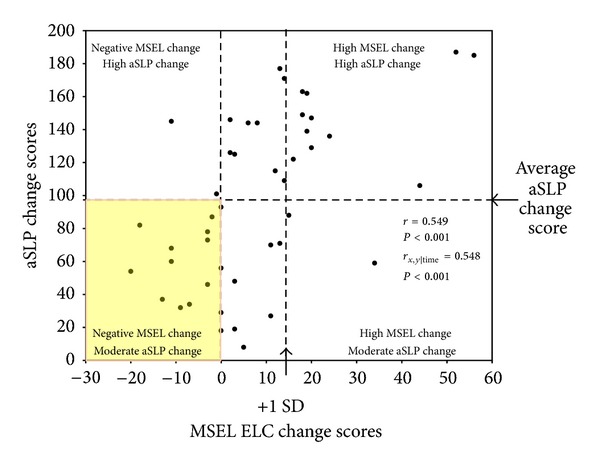
Correlation between MSEL ELC change scores and aSLP change scores (i.e., change in scores from intake to exit). Correlation coefficients are reported for the Pearson correlation and partial correlation controlling for the amount of time each child was in treatment. A significant positive correlation was found. Children exhibited an average of 97 aSLP skills learned on average and an average change score of 8 on the MSEL ELC.

**Figure 4:**
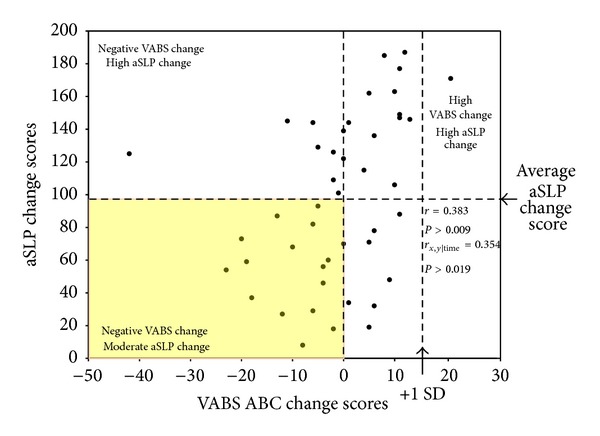
Correlation (controlling for the amount of time each child was in treatment) between VABS ABC change scores and aSLP change scores (i.e., change in scores from intake to exit). Correlation coefficients are reported for the Pearson correlation and partial correlation controlling for the amount of time each child was in treatment. A significant positive correlation was found. Children exhibited an average of 97 aSLP skills learned on average and an average change score of −1 on the VABS ABC.

**Figure 5:**
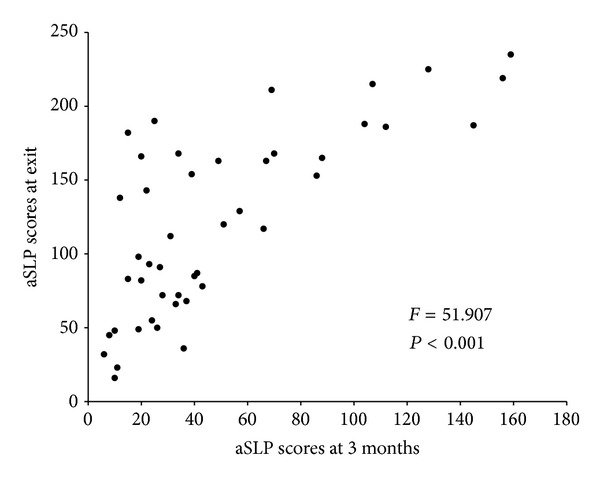
A linear regression between aSLP scores at 3 months and exit. aSLP scores at 3 months into treatment significantly predicted aSLP scores when participants exited the program.

Partial correlations between the MSEL, VABS, and aSLP scores, controlling for the number of months the children were in the treatment program, were conducted. Since child age or time passing is not controlled for on the aSLP as it is not a standardized measure, the effect of the amount of time the child spent in treatment on aSLP scores was investigated. The amount of time the child was in treatment had a small negative correlation with the aSLP scores at intake (*r* = −0.332, *P* = 0.026) and a significant correlation with aSLP change scores (*r* = 0.422, *P* < 0.005). Time in treatment was not correlated with aSLP scores at exit, or scores on the MSEL or VABS at any time point. It appears that the amount of time the child was in treatment may influence aSLP scores and therefore may impact the relationship observed between the aSLP and the MSEL and VABS. Thus, partial correlations were conducted holding the amount of time in treatment constant to adjust for the effect time in treatment may have on the aSLP, MSEL, and VABS. aSLP scores at intake were positively correlated with the MSEL ELC at intake (*r* = 0.580, *P* < 0.001) and the VABS ABC at intake (*r* = 0.654, *P* < 0.001; see Figure 2). aSLP scores at exit were positively correlated with the MSEL ELC (*r* = 0.713, *P* < 0.001) and VABS ABC (*r* = 0.643, *P* < 0.001) at exit (see Figure 2). aSLP change scores were also positively correlated with MSEL ELC change scores (*r* = 0.548, *P* < 0.001) and VABS ABC change scores (*r* = 0.354, *P* = 0.019; see Figures 3 and 4). Notably, 33% of the sample (i.e., 15 toddlers) showed a decrease or no gain in their MSEL ELC and 36% of the sample showed a decrease or no gain in their VABS ABC scores, while the aSLP captured a skill gain for these children that averaged a change of 97 (*r* = 8–187) newly learned skills (see Figures 3 and 4). A linear regression was conducted to evaluate whether aSLP scores at three months into the intervention program predicted aSLP scores when the participants exited the program. The results of the regression indicated aSLP scores at three months into intervention explained 54.8% of the variance (*R* ^2^ = 0.547, *F*(1,43) = 51.907, *P* < 0.001; see Figure 5). aSLP scores at three months into intervention significantly predicted aSLP scores at exit (*β* = 0.72, *P* < 0.001).

Two of the children displayed very high scores across all three assessments at intake. These children were the only participants to score two standard deviations above the mean score on at least two of the three assessments. Therefore, the following analyses also were conducted excluding these two participants. Partial correlations controlling for the number of months the children were in treatment were conducted. aSLP scores at intake were positively correlated with the MSEL ELC at intake (*r* = 0.336, *P* = 0.030) and VABS ABC at intake (*r* = 0.440, *P* = 0.004). aSLP scores at exit were positively correlated with the MSEL ELC (*r* = 0.738, *P* < 0.001) and VABS ABC (*r* = 0.684, *P* < 0.001) at exit. aSLP change scores were also positively correlated with MSEL ELC change scores (*r* = 0.648, *P* < 0.001) and VABS ABC change scores (*r* = 0.585, *P* < 0.001). A linear regression was conducted to evaluate whether aSLP scores at three months into the intervention program predicted aSLP scores when the participants exited the program. aSLP scores at three months into intervention explained 47% of the variance (*R* ^2^ = 0.470, *F*(1,41) = 36.360, *P* < 0.001). aSLP scores at three months into intervention significantly predicted aSLP scores at exit (*β* = 0.686, *P* < 0.001).

## 4. Discussion

The results of this study provide support for the supplemental use of a curriculum-based assessment, the aSLP, for determining the benefits of an early intervention program for children with ASD. While standardized assessments such as the MSEL and the VABS are valuable tools in evaluating child outcome, they have some limitations that suggest the need for the addition of a curriculum-based measure such as the aSLP. High variability in scores among participants was seen across all assessments. Assessments were highly correlated, likely indicating that children who had higher overall cognitive and adaptive functioning were more likely to have mastered more skills, which is not surprising but may support the validity of a curriculum-based assessment. The VABS standard scores were not sensitive to change in the children's skills during intervention as evidenced by nonsignificant changes in VABS standard scores over time. The MSEL and aSLP showed significant changes in scores over time. This may indicate that the aSLP and MSEL are sensitive to change in skill acquisition and development but do not capture change in adaptive functioning. One explanation may be that the STAR program is more heavily focused on behaviors related to cognitive gains for these very young children. Alternatively, the children may be showing specific changes in skill acquisition during treatment and testing sessions, but these skills may not generalize to daily functioning and use across multiple environments. The assessments may simply be measuring very different skills, or the age of the children may be a factor given the limited change in adaptive behavior expected on the VABS at this young age.

Given that the children's scores on the aSLP were positively correlated with the MSEL and VABS at all time points and over time, it is likely that the aSLP is a comparable measure of child progress despite its lack of standardized norms. Two participants displayed high scores across all three assessments at intake. Correlations between the aSLP and MSEL and VABS were stronger with these participants included; however, intake, exit, and change scores were still moderately correlated when these participants were removed from the analyses. The evidence of significant positive correlations with or without these two participants suggests that the extreme scores of these two participants were not driving the correlations between the assessments and rather supports the use of the aSLP across a wide range of children with varying levels of functioning.

The addition of the aSLP in this study was very informative, in terms of ongoing child response to treatment, treatment trajectory, and overall outcome at the conclusion of the study. For example, over 1/3 of the children showed a decrease or no gain in MSEL or VABS standardized scores. However, all children showed an increase in skills mastered as measured by the aSLP. Tracking progress of children can become complicated when utilizing standardized measures as standardized scores are determined by comparing raw scores on the assessment to norms of children of the same chronological age. Thus, when evaluating children who may be progressing slower than typically expected, those children may actually exhibit no gain or a decrease in standardized scores since the children are not keeping up with the expected progress. Thus, interpreting the treatment success for this particular group of children based on the MSEL or VABS alone makes it difficult to determine rate of progress. It is important to have a measure of treatment progress in children who are developing more slowly to make appropriate changes to intervention strategies, determine rate of learning, and make predictions regarding service needs.

Additionally, the aSLP allowed the children's in-home treatment coordinators to quickly and easily determine the children's intervention progress throughout the intervention period. The results of the aSLP provided detailed information about specific behaviors, rather than general information about the child's ability level, which is provided by standardized assessments. This allowed for easy analysis of child functioning at the skill by skill level, which is particularly beneficial for treatment planning. Very young children, like those in the current study, have very little experience with testing and may not do well on standardized tests, which makes using them to guide early treatment even more problematic. In contrast, the aSLP may be useful for early goal development and allow for a systematic yet individualized process for the child's treatment program to follow. Additionally, the aSLP provided some information to hypothesize child's trajectory of treatment. aSLP scores after 3 months of treatment predicted aSLP scores at exit. The aSLP scores provide information early on regarding the child's predicted treatment trajectory. Early information on responsivity to treatment may provide valuable information for individualization of treatment. The utility of the aSLP for assessing and detecting treatment trajectory information so early on gives it a unique advantage for practical use within community settings, beyond what a standardized assessment might provide. Information about child progression through a curriculum such as the aSLP can be a very useful complement to standardized assessments as it gives a researcher or practitioner the opportunity to observe the real-world effects of treatment.

### 4.1. Limitations

The main limitation of the current study is that the in-home coordinator for each child administered the aSLP, rather than employing a blind rater to assess the children. It is possible that the in-home coordinators had an expectancy bias when assessing the children, which may have influenced the scores derived from the aSLP. However, the in-home coordinators were not providing direct service to the children. Children were familiar with them but did not receive intervention directly from the person doing the testing. Blind raters were not employed due to the amount of additional resources that would be needed to carry out a blind rating system and because the purpose of the aSLP was to provide clinical data. Another limitation was the lack of formalized procedures to ensure interrater reliability of the aSLP ratings between in-home coordinators. Although the scoring procedure is straightforward and in-home coordinators were trained in the same manner, there is a potential for differences to occur between aSLP raters. Another concern with the use of the aSLP over time is the possibility of practice effects. Specific steps were taken to try to limit practice effects. The aSLP was given only every three months and tested items not specifically targeted during treatment. Feedback was not provided to the children regarding appropriate or correct responses. The task format was similar to that of intervention and familiar procedures tend to lead to fewer practice effects. However, it remains possible that practice effects may account for some of the increase in skills measures on the aSLP.

Additional limitations include the use of a small, young sample. Forty-five children under the age of three were evaluated throughout participation in an early intervention program. Future research should evaluate the usefulness of a curriculum-based measure such as the aSLP in a larger and older population. Likely, the benefits of a curriculum-based measure would generalize across populations, as the same benefits of ease of implementation, frequency of use, and direct translation to informing programming would be applicable. Additionally, as the aSLP is not a normed, standardized measure, we do not have a good understanding of the validity and reliability of the measure beyond what was explored in the current study. As such, the aSLP provides limited information in regard to the functioning level of the child with respect to same-aged peers and rather focuses on the skills learned in the treatment program. Therefore we are suggesting the use of a curriculum based assessment in conjunction with, not as a replacement for, standardized assessments. Also, although the items on the aSLP were not directly taught, the aSLP was used to develop treatment goals which may have inflated progress. However, skill gain was the important variable for these analyses. Additionally, these limitations are generalizable to how the assessment would be used in community practice; therefore this study represents an examination of the utility of the measure in community care.

### 4.2. Future Research

The aSLP shows promise as a useful tool for measuring intervention progress and assisting with intervention development; however future research is needed to fully determine accuracy and limitations of the measure. Future studies should compare progress across groups of children in various intervention programs, including those not using the aSLP to develop curriculum items, to examine sensitivity to specific treatment changes. This will also help reduce issues of children learning items that are directly related to the assessment. Studies of the psychometric properties of the aSLP are imperative. Future research should establish interrater reliability with blind raters to further evaluate the rigor of this type of measure. Examination of the generalization of skills assessed by the aSLP is needed, including the relationship between the specific items on the aSLP and those on standardized measures of adaptive and cognitive functioning.

### 4.3. Summary

One of the greatest challenges facing early intervention researchers and community providers today is finding accurate and useful methods for assessing child response to treatment and overall outcome during and after a course of early intervention [33]. There is a large amount of money spent on early intervention and this spending is likely to increase over time. As such, it is important to have a recommended set of assessments that effectively measure child functioning and progress during early intervention [34]. In addition, with the increased responsibility of insurance companies over the coming years, use of the aSLP may offer assistance to ASD service providers who are challenged with providing information on child response to treatment. ASD service providers will most likely need information about child progress, treatment planning, and whether an intervention program is beneficial to the child overall. Standardized cognitive and adaptive behavior assessments are invaluable but the addition of a detailed curriculum-based measure that can be repeated often throughout intervention is critical to both obtaining an accurate measure of a child's abilities and being able to quickly and effectively keep up with the child's progress in a way that maximizes treatment benefit for the child. Curriculum-based measures such as the aSLP may allow providers to gather all these pieces of information on a regular basis. Thus while standardized assessments such as the MSEL and the VABS are valuable tools in evaluating child outcome, the results of this study provide support for the additional use of a curriculum-based measure, the aSLP, for determining the benefits of an early intervention program for children with ASD.

## Figures and Tables

**Table 1:** Participant demographics.

Gender	
Male	36
Female	9
Age (months)	
Intake	22.67 (4.13)
Exit	34.06 (3.25)
Length of treatment program	
Months in intervention	11.53 (4.04)
Ethnicity	
Hispanic or Latino	12
Not-Hispanic or Latino	21
Not reported	12
Race	
American Indian or Alaska Native	0
Asian	3
Black or African American	4
Native Hawaiian or other Pacific Islander	1
White	25
Not reported	12

Note: averages listed with standard deviations within parentheses.

**Table 2:** aSLP assessment example.

Lesson	Concept	Example instruction cue	Target skill	Student response		
Preverbal communication	Goal directed reach to request	Teacher holds up object	Child reaches toward desired object	Rarely	Sometimes	Usually
	Eye contact to request	Teacher blocks access/withholds desired object	Child makes eye contact to obtain desired object	Rarely	Sometimes	Usually
	Proximal point to request	Teacher holds up object	Child points to desired object	Rarely	Sometimes	Usually
		Teacher holds up two objects	Child points to indicate a choice between two objects	Rarely	Sometimes	Usually

Expanded learning to play	Multiple-step imitation	Verbal cue: “Do this.” Nonverbal play model: teacher puts man in toy car and then pushes the car	Student models teacher's action (e.g., student puts man in a toy car and then student pushes car)	Never	If prompted	Independently

Expanded playing with toys	Following two- or three-step play commands	Teacher gives verbal cue (e.g., “Put man in car and push car”)	Student responds to teacher cue without need for initiation (e.g., student puts man in the toy car and pushes it)	Never	If prompted	Independently
	Independent construction or functional play	No specific cue is needed	Child plays appropriately with toy during reinforcement phase of PRT lessons	Never	If prompted	Independently

**Table 3:** Summary of assessment scores.

MSEL ELC	
Intake	75.44 (14.87)
Exit	83.51 (21.76)*
Change scores	8.07 (16.46)
VABS ABC	
Intake	84.38 (11.46)
Exit	83.13 (10.85)
Change scores	−1.24 (11.58)
aSLP score	
Intake	23.56 (24.27)
Exit	120.58 (61.87)*
Change scores	97 (50.96)

Note: average scores listed with standard deviations within parentheses.

*Indicates statistically significant change from intake to exit.
